# The TiO_2_-ZnO Systems with Multifunctional Applications in Photoactive Processes—Efficient Photocatalyst under UV-LED Light and Electrode Materials in DSSCs

**DOI:** 10.3390/ma14206063

**Published:** 2021-10-14

**Authors:** Adam Kubiak, Sonia Żółtowska, Aleksandra Bartkowiak, Elżbieta Gabała, Natalia Sacharczuk, Maciej Zalas, Katarzyna Siwińska-Ciesielczyk, Teofil Jesionowski

**Affiliations:** 1 Institute of Chemical Technology and Engineering, Faculty of Chemical Technology, Poznan University of Technology, Berdychowo 4, PL-60965 Poznan, Poland; sonia.zoltowska-aksamitowska@doctorate.put.poznan.pl (S.Ż.); katarzyna.siwinska-ciesielczyk@put.poznan.pl (K.S.-C.); 2 Institute of Chemistry and Technical Electrochemistry, Faculty of Chemical Technology, Poznan University of Technology, Berdychowo 4, PL-60965 Poznan, Poland; 3 Faculty of Chemistry, Adam Mickiewicz University, Uniwersytetu Poznańskiego 8, PL-61614 Poznań, Poland; aleksandra.bartkowiak@amu.edu.pl (A.B.); natsac@st.amu.edu.pl (N.S.); maciej.zalas@amu.edu.pl (M.Z.); 4 Institute of Plant Protection, National Research Institute, Węgorka 20, PL-60318 Poznań, Poland; e.gabala@iorpib.poznan.pl

**Keywords:** TiO_2_-ZnO system, hydrothermal synthesis, photocatalysis, photo-oxidation process, DSSC

## Abstract

The main goal of the study was the hydrothermal-assisted synthesis of TiO_2_-ZnO systems and their subsequent use in photoactive processes. Additionally, an important objective was to propose a method for synthesizing TiO_2_-ZnO systems enabling the control of crystallinity and morphology through epitaxial growth of ZnO nanowires. Based on the results of X-ray diffraction analysis, in the case of materials containing a small addition of ZnO (≥5 wt.%), no crystalline phase of wurtzite was observed, proving that a high amount of modified titanium dioxide can inhibit the crystallization of ZnO. The transmission electron microscopy (TEM) results confirmed the formation of ZnO nanowires for systems containing ≥ 5% ZnO. Moreover, for the synthesized systems, there were no significant changes in the band gap energy. One of the primary purposes of this study was to test the TiO_2_-ZnO system in the photodegradation process of 4-chlorophenol using low-power UV-LED lamps. The results of photo-oxidation studies showed that the obtained binary systems exhibit good photodegradation and mineralization efficiency. Additionally, it was also pointed out that the dye-sensitized solar cells can be a second application for the synthesized TiO_2_-ZnO binary systems.

## 1. Introduction

Recently, the continuous development of innovative technologies placed more and more demands on many scientific fields, including material engineering. Increasingly stringent requirements are imposed on the materials obtained to implement them in industrial applications [1]. One of the most popular metal oxides is titanium dioxide, which has several possible applications, e.g., in water treatment processes [2], solar cells (dye sensitized solar cell—DSSC) [3], and lithium-ion batteries [4]. Therefore, many research groups around the world work on methods for its modification. One of the most popular is the preparation of hetero-structural oxide systems with ZnO. It is one of the curious oxides due to its properties, such as semiconductors materials (n-type), as well as large exciton binding energy (60 mV) [5]. The combination of the properties of both oxides mentioned above has been used, among others, by Patwari et al. [6]. They used the heterostructures systems as the working electrode in photovoltaic devices. Their research groups’ results also suggest that electrodes containing ZnO work more efficiently than pure TiO_2_ ones [7].

Despite the evolution of materials science, it should be noted that development is also necessary for the field of technologies used in the studied applications. The issue above is evident in the example of the photoactive processes. When analyzing the phenomenon of photocatalysis, it should be noted that many research teams work on new materials and their modification to use them in the processes of degradation of the water sewage [8,9]. However, other critical aspects of photooxidative efficiencies, such as the type of light source and its power, are overlooked in the research [10]. Two main types of lighting are used in photodegradation processes: UV and visible or solar light [11]. It is evident that the most advantageous from the point of view of availability is sunlight. However, its implementation in wastewater treatment systems seems limited due to the high cost and a large area for installation. Likewise, the efficiency of the reactor depends upon the direction, intensity, and availability of solar light [12,13]. Nevertheless, the use of classical ultraviolet (UV) sources is limited for several reasons. The first reason is the harmful side effect of UV sources and high voltage at the initial stage and cooling requirement [14]. However, the main issue is the usage of hazardous mercury, classified by the U.S. Environmental Protection Agency as air pollutants (HAP) and very harmful to human health [15]. The new and energy-efficient alternative is ultraviolet light-emitting diodes (UV-LED), meeting the needs of the described difficulties associated with conventional UV light sources [16]. One of the main advantages of LEDs over traditional light sources is their lifetime. The light-emitting diodes are semiconductor p–n junction devices, including gallium arsenide, gallium arsenide phosphide, gallium phosphide, or indium gallium nitride. In the case of LED, the flow of current is one-directional and emits ultraviolet light in a narrow spectrum in the form of electroluminescence [17]. In addition, LEDs are less energy-consuming, thus generating less heat and, therefore, do not require additional cooling. Furthermore, most importantly, UV-LEDs are cheaper than mercury or xenon lamps. They are available and commercially used for various applications, e.g., fluorescent detection of fraudulent documents, forensic investigations, and disinfecting devices [18]. However, LED-based solutions also have some disadvantages, such as the limited view angle of these emission sources and heterogeneous light distribution. Therefore, considering using LED sources in photoactive processes, it is necessary to select an appropriate material. Hence, it should be noted that the focus on only one field (e.g., materials science) is insufficient [19].

On this basis, the main assumptions of this work were created. The main novelty aspect of the work concerns the hydrothermal-assisted synthesis method enabling the control of crystallinity and morphology through epitaxial growth of ZnO nanowires. According to Sugunan et al. [20], the amines can play the role of non-polar chelating agent in the synthesis of ZnO crystals. Thus, it can determine the preferred epitaxial growth. Therefore, in our work, the first step in the synthesis of TiO_2_-ZnO binary systems was to obtain titanium dioxide modified with triethylamine, which, in the next stage, has played the role of growth determining factor of ZnO structures. An additional aspect of the novelty is focused on applying cheap LED SMD technology to construct low-power UV-LED photoreactors. Consequently, the resulted systems were used to produce light in the degradation of 4-chlorophenol. The performed photo-oxidation tests proved that the heterostructure materials demonstrate high photocatalytic activity in the removal of 4-chlorophenol. Furthermore, TiO_2_-ZnO systems, apart from high photoactivity, can be characterized by suitable photovoltaic parameters as the working electrode in DSSC cells, and such a multifunctional approach is not common in the available scientific literature.

## 2. Materials and Methods

### 2.1. Materials

Titanium tetrachloride (97%, TiCl_4_; Sigma-Aldrich, St. Louis, MO, USA), zinc acetate dihydrate (p.a., Zn(CH_3_COO)_2_*2H_2_O; Sigma-Aldrich, St. Louis, MO, USA), triethylamine (>97%, C_6_H_15_N; Sigma-Aldrich, St. Louis, MO, USA), sodium hydroxide (p.a., POCh, Gliwice, Poland) ethylcellulose (p.a., Sigma-Aldrich St. Louis, MO, USA), α-terpineol (96%, Sigma-Aldrich, St. Louis, MO, USA), 1-propyl-3-methyl-imidazole iodide (97%, Sigma-Aldrich, St. Louis, MO, USA), 4-tert-butylpiridine (98%, Sigma-Aldrich, St. Louis, MO, USA), acetonitrile (LC-MS ultra-pure, Honeywell, New Jersey, USA), acetic acid (p.a., POCh, Gliwice, Poland), ammonia 25% solution (p.a., POCh, Gliwice Poland), anhydrous ethanol (99.8%, POCh, Gliwice Poland), iodine (pure, POCh, Gliwice, Poland), N3 dye (pure, Ruthenizer 535, Solaronix, Aubonne, Switzerland), H_2_PtCl_6_ (99.9% Merck, Darmstadt, Germany), guanidine thiocyanate (97%, Fluka, Buchs, Switzerland), ionomeric foil Meltonix (Solaronix, Aubonne, Switzerland), and potassium dihydrogen phosphate (p.a., Sigma-Aldrich, St. Louis, MO, USA) were used. All reagents were of analytical grade and used without any further purification. The water used in all experiments was deionized.

### 2.2. Synthesis of TiO_2_-ZnO Systems

The synthesis methodology of TiO_2_-ZnO systems was performed in two steps. The first stage was focused on the synthesis of TiO_2_. The 100 cm^3^ of the primarily prepared 1% aqueous solution of titanium tetrachloride was put in a reactor and homogenized on a magnetic stirrer (IKA Werke GmbH, Staufen im Breisgau, Germany). The triethylamine solution (>97%) was added until the solution’s pH reached 9. Hence, the prepared mixture was transferred to an autoclave (Parr Instrument Co., Moline, IL, USA) and subjected to a hydrothermal treatment for 12 h at 160 °C. The second stage involved a synthesis of the TiO_2_-ZnO systems. Firstly, the proper amount of zinc acetate was dissolved in 50 cm^3^ of deionized water and stirred. At the same time, the titania obtained in the first stage was sonicated (1 g TiO_2_ in 10 cm^3^ deionized water) (Sonic-3 ultrasonic cleaner, Polsonic, Poland). Thereafter, it was added to the solution of zinc acetate and further mixed for 0.5 h. Next, the 1 M solution of sodium hydroxide was added till the mixture reached pH 8. Then, the mixture was transferred to an autoclave (Parr Instrument Co., Moline, IL, USA) and treated hydrothermally in the same conditions as before. The synthesized TiO_2_-ZnO systems were washed and filtered with distilled water and ethanol and dried for 6 h at 60 °C. Five oxide systems with the different amounts (wt.%) of ZnO were synthesized and described as TiO_2_-(2.5%)ZnO, TiO_2_-(5%)ZnO, TiO_2_-(10%)ZnO, TiO_2_-(15%)ZnO, TiO_2_-(20%)ZnO, and also, two reference oxide samples were obtained and labeled as TiO_2_ and ZnO, respectively.

### 2.3. Characteristics of Synthesized TiO_2_-ZnO System

The elemental contents of N, C, H, and S of modified TiO_2_ samples were measured using a Vario EL Cube instrument (Elementar Analysensysteme GmbH, Langenselbold, Germany). The samples were placed in the device and combusted in an oxygen atmosphere. After passing through appropriate materials in a helium stream, the resulting gases were separated in an absorption column.

The X-ray Photoelectron Spectroscopy (XPS) analyses were recorded on Specs UHV spectrometer (SPECS GmbH, Berlin, Germany) with a charge neutralizer. As the reference to rectify the binding energies was used, the C 1s peak at 284.8 eV.

Characteristic functional groups on the surface of the TiO_2_ sample were identified using Fourier transform infrared spectroscopy (FTIR) working in transmission mode. The measurement was performed using a Vertex 70 spectrometer containing the LMCT detector (Bruker, Bremen, Germany). The FTIR spectra were obtained in the transmission mode between 4000 cm^−1^ and 420 cm^−1^. The efficiency of the modification of TiO_2_ with the use of triethylamine was verified through attenuated total reflectance (ATR) spectroscopy. This analysis was conducted using a single-reflection diamond ATR accessory (Platinum ATR, Bruker Optics GmbH, Bremen, Germany). The TiO_2_-ZnO systems were measured in the classical transmission mode, using potassium bromide (KBr) as the background.

The X-ray diffraction method was carried out to determine the crystal structure of the obtained samples. The analyzer Rigaku Miniflex 600 (Rigaku, Tokyo, Japan) operating with Cu Kα radiation (α = 1.5418 Å) was applied. The patterns were obtained over an angular range of 20–80°. The diffraction patterns were evaluated by the Rietveld method using the Fullprof software [21]. The crystallite size of the synthesized samples in the vertical direction to the corresponding lattice plane was determined using Scherrer’s equation [22,23] with a constant equal to 0.891.

The morphology of the obtained samples was determined using MIRA3 scanning electron microscope (TESCAN, Brno, Czech Republic) and Hitachi HT7700 (Hitachi, Tokyo, Japan) transmission electron microscope working in high contrast and high-resolution mode.

The EDX (during TEM analysis) and EDXRF analyses were performed to determine the surface composition. The maps of titanium and zinc elements of TiO_2_-ZnO systems were performed using the Hitachi HT7700 (Hitachi, Tokyo, Japan) operating in the STEM mode with a system to the energy dispersive X-ray microanalysis (Thermo Scientific, Waltham, MA, USA). The content of appropriate oxides was determined using an Epsilon4 EDXRF spectrometer (PANalytical, Malvern, UK).

The DRS analysis was carried out using a Thermo Scientific Evolution 220 (Thermo Scientific, Waltham, MA, USA) spectrophotometer equipped with a PIN-757 integrating sphere. The bandgap energies of the obtained samples were calculated based on the Kubelka–Munk function:

(1) $$F\left(R\right)=\frac{{\left(1-R\right)}^{2}}{2R}$$

where *R* is reflectance, which is proportional to the absorption of radiation, by plotting:

(2) $$F{\left(R\right)}^{0.5}E_{ph}^{0.5}$$

where *E_ph_* means the photon energy.

Jupiter STA 449F3 apparatus (Netzsch, Selb, Germany) was applied to perform the thermogravimetric analysis (TGA/DTG). The thermal stability tests were carried out under flowing nitrogen (20 cm^3^/min) at a heating rate of 10 °C/min over a temperature range of 25–1000 °C, with an initial sample weight of approximately 10 mg.

### 2.4. Photocatalytic Activity of TiO_2_-ZnO Systems

**Figure 1:**
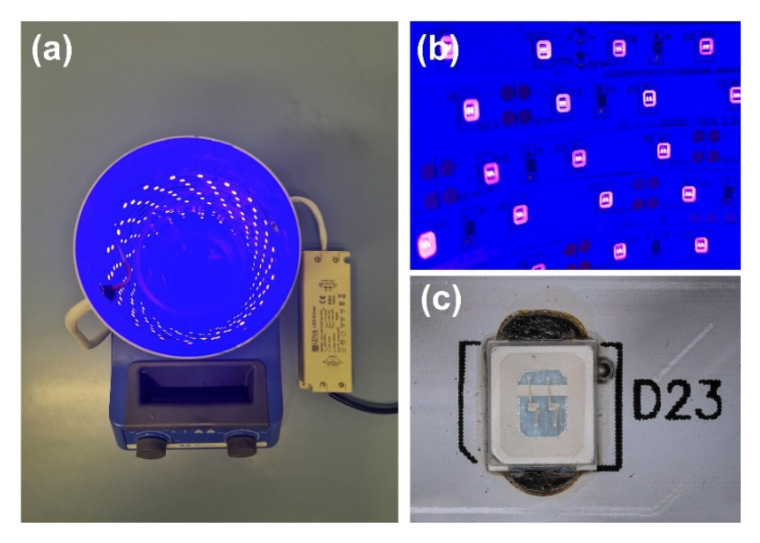
(**a**) UV-LED photocatalytic reactor, (**b**) UV-LED strip light source, and (**c**) focus on single SMD diode.

The synthesized TiO_2_-ZnO systems’ photo-oxidation activity was evaluated in the degradation process of a model organic impurity—4-chlorophenol. The study used the UV-LED light source, based on the LED strips with wavelength of 395 nm. A low-voltage LED strip—2835 SMD (ELED, Klucze, Poland), containing 60 LEDs/m, and generating power of 7.2 W/m, was used to construct the reactor. The 1.4 m and 2.8 m of the above-described LED strip were used to obtain rectors with a specific power (10 and 20 W), respectively, which were placed inside the pipe covered with aluminum tape to dissipate the generated heat better. Finally, the resulting system was connected to the ballast (Lena Lighting, Sroda Wielkopolska, Poland), and the obtained power was confirmed, which was in line with the assumed value. The resulting LED photoreactor is shown in Figure 1. Initially, 100 cm^3^ of the 4-chlorophenol and 100 mg of the photocatalyst were introduced into the reactor. The resulting suspension was homogenized using a magnetic stirrer (IKA Werke GmbH, Staufen, Germany) in darkness (30 min) to establish adsorption/desorption equilibrium. Next, the UV-LED lamp was switched on, and the reaction mixture was irradiated. Every 20 min (up to 160 min, then we stopped the irradiation), 3 cm^3^ of the suspension was collected and filtered through a syringe filter (Macherey-Nagel, Duren, Germany). The filtrate was analyzed using a UV-Vis spectrophotometer (V-750, Jasco, Tokyo, Japan) in the 200–700 nm wavelength range, using the demineralized water spectrum as a baseline. The maximum absorbance of pollution at wavenumber 280 nm was observed. The photocatalytic activity of the samples was determined by applying a calibration curve method about the formula *y* = 0.01*x* + 0.277, where *x* was the 4-chlorophenol concentration, and *y* was the maximum absorbance value.

Additionally, the total organic carbon (TOC) was carried out using a TOC-L analyzer (Shimadzu, Tokyo, Japan) to determine the mineralization efficiency.

### 2.5. Photovoltaic Properties

#### 2.5.1. DSSC Preparation

The already described method has been used to prepare the viscous pastes of TiO_2_-ZnO systems [24,25]. The pastes containing selected TiO_2_-ZnO materials, terpineol, and ethylcellulose have been used to prepare working electrodes by spreading the pastes on fluorine-doped tin oxide glass (FTO) substrates using the “*doctor-blade*” technique. Freshly prepared electrodes have been annealed in the oven at 450 °C for 2 h, and after cooling down, the working electrodes were immersed in 40 mM TiCl_4_ aqueous solution and kept at 70 °C for one hour. Next, the electrodes have been washed with water and ethanol, dried, and annealed in the oven at 450 °C for 30 min. The electrodes cooled down to approximately 80 °C, then immersed in the 10^−4^ M ethanolic solution of N3 sensitizing dye, and were kept 24 h in the dark. The dye loading has been determined using our previously published procedure [25]. In the meantime, the Pt coated FTO counter electrodes have also been prepared using the procedure described by us elsewhere [26]. The working and counter electrodes have been used to assemble the sandwich-type device using the Surlyn ionomeric hot-melted foil (thickness 25 µm) as a sealant and spacer between the electrodes. Finally, the liquid electrolyte containing 0.6 M of 1-propyl-3-methyl-imidazolium iodide, 0.03 M of iodine, 0.1 M of guanidine thiocyanate, and 0.5 M of 4-*tert*-butylpiridine in acetonitrile was injected into the cells through the two holes predrilled in the counter electrodes. Then, the cells were sealed with the piece of Surlyn foil and microscope cover slides. The typical active area of the obtained DSSC was approximately 0.125 cm^2^.

#### 2.5.2. DSSC Characterization

The photovoltaic properties of the cells were measured under irradiation using a Sun 2000 class A solar simulator (ABET Technologies, Milford, CT, USA). The simulator was equipped with an AM 1.5G filter, with the light intensity was adjusted to 100 mW/cm^2^ using a silicon reference cell equipped with a KG5 filter (ReRa Systems, Nijmegen, The Netherlands). J–V curves were recorded using a Gamry Interface 1000 Potentiostat/Galvanostat/ZRA (Gamry Instruments, Warminster, PA, USA).

Electrochemical impedance spectra were recorded under standard AM 1.5G simulated solar irradiation, using the Sun 2000 class A solar simulator (ABET Technologies, Milford, CT, USA). The frequency was measured using a Gamry Interface 1000 Potentiostat/Galvanostat/ZRA (Gamry Instruments, Warminster, PA, USA), ranging from 0.1 Hz to 100 kHz, under VOC bias conditions V_AC_ = 10 mV.

## 3. Results and Discussion

### 3.1. Results of Titanium Dioxide Modified with Triethylamine

First, steps were taken to confirm the effective modification of the titania. For this purpose, elementary analysis, Fourier transform infrared spectroscopy, and X-ray photoelectron spectroscopy were performed.

In the first stage of characterization of titanium dioxide modified with triethylamine, elemental analysis was carried out. The obtained results were collected and are presented in Table 1.

Based on the obtained results of elemental analysis, it was observed that for the sample of TiO_2_ modified with triethylamine, a significant content of nitrogen, carbon, and hydrogen was noted. In the case of nitrogen, the content was 1.8%, for carbon 3%, and for hydrogen 2%. An apparent increase in the content of the elements mentioned above may indicate an effective modification process.

However, to confirm the effective modification of TiO_2_, the next step was to analyze the chemical states of the elemental components using the XPS technique.

**Figure 2:**
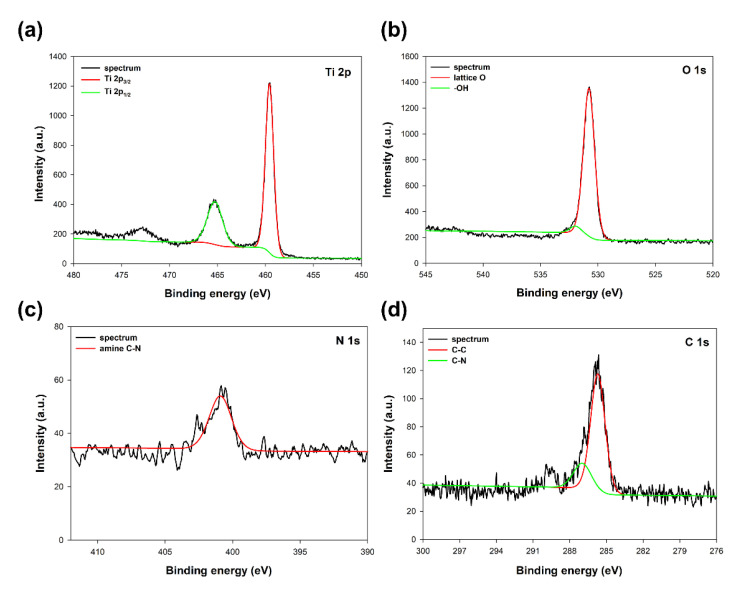
XPS spectra of specific regions: (**a**) Ti 2p, (**b**) O 1s, (**c**) N 1s, and (**d**) C 1s for TiO_2_ modified with triethylamine.

As was expected, titanium, oxygen, nitrogen, and carbon have been observed in the spectra of the analyzed sample. The selected high-resolution XPS spectra of specific regions, Ti 2p, O 1s, N 1s, and C 1s, respectively, are presented in Figure 2.

The Ti 2p region at the TiO_2_ sample spectrum (see Figure 2a) exhibited two characteristic peaks at binding energies 459.6 eV and 465.2 eV, associated with Ti 2p3/2 and Ti 2p1/2 of Ti^4+^ ions in oxide lattice, respectively [27]. The O 1s region (see Figure 2b) on the spectrum shows a double peak, which, after deconvolution, may be resolved to the lattice oxygen and adsorbed surface –OH groups [28]. The positions of the O 1s were 530.7 eV for lattice O and 532.1eV for –OH groups. In the N 1s region (see Figure 2c), the peak characteristic for the amine C-N bond at a binding energy of 400.8 eV may be observed [29]. The C 1s region (see Figure 2d) shows two characteristic peaks centered at binding energies 286.9 eV and 285.7 eV, corresponding to C-C and C-N bonds derived from the triethylamine used [30]. However, no shift of the peaks in the Ti 2p region, also no additional peaks may be observed on the spectrum, which indicates that the addition of modifier (TEA) is not disturbing the TiO_2_ lattice, and the modification only takes place on the surface of the titanium dioxide [31]. On the other hand, the presence of nitrogen and carbon in the analyzed TiO_2_ sample indicates an effective modification process with triethylamine.

**Figure 3:**
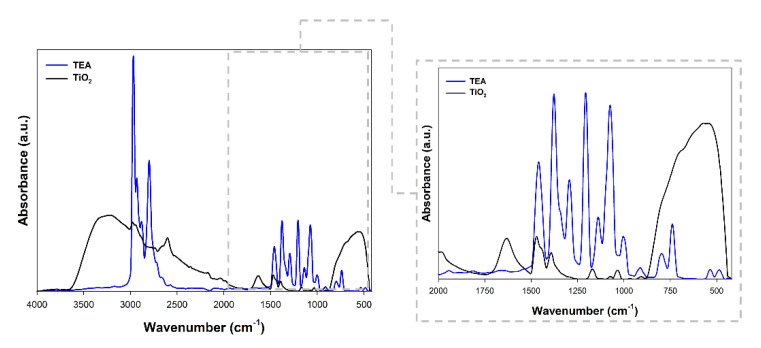
FTIR spectra for triethylamine (TEA) and TiO_2_ modified with TEA.

Fourier transform infrared spectroscopy was performed to confirm the effective modification of titanium dioxide. The obtained spectra are presented in Figure 3.

Figure 3 shows the FTIR spectra of the TEA and TiO_2_ sample. Interestingly, the C–H stretching peaks are attributed at 1391 cm^−1^ and 2971 cm^−1^, which may evidence the effective modification of titanium dioxide with triethylamine [32]. Additionally, the band at 1381 cm^−1^ was referenced as the C-N stretching vibration was found [33]. The peak obtained for the modified material at 1461 cm^−1^ corresponded to the ethyl groups in the tertiary amine [34]. In addition to the bands characteristic of triethylamine, the stretching vibrations of the ≡Ti-O (750 cm^−1^), as well as stretching vibrations of the hydroxyl group (3500 cm^−1^) and band from physically adsorbed water (1600 cm^−1^) [9] were noted.

On the basis of the available scientific literature related to the use of triethylamine as a modifier of oxide materials, it should be noted that stretching vibrations of the C-H groups (1390 cm^−1^) are indicated as the main band indicating effective modification [35]. Moreover, Liu et al. [36], showed that in the materials modified with TEA one can observe the disappearance of the band at 1248 cm^−1^, which is caused by the interactions between the base material and the modifier. Similar observations to those presented in our work were described by Haque and Mahalakshmi [37], who received zinc oxide containing triethylamine. As the key bands indicating the modification, the authors indicated bands at the wavenumber of 1023 cm^−1^, 1400 cm^−1^. On the other hand, Liu et al. [38] pointed out that in the materials modified with TEA, the intensity of the bands characteristic may be reduced.

Based on the above results and scientific knowledge, the effectiveness of TiO_2_ modification with the use of triethylamine was confirmed.

### 3.2. Results for TiO_2_-ZnO Systems

In the next stage, a comprehensive physicochemical analysis of the obtained binary systems was carried out to determine their crystallographic and morphological parameters, surface composition, and thermal stability.

#### 3.2.1. Crystal Structure

**Figure 4:**
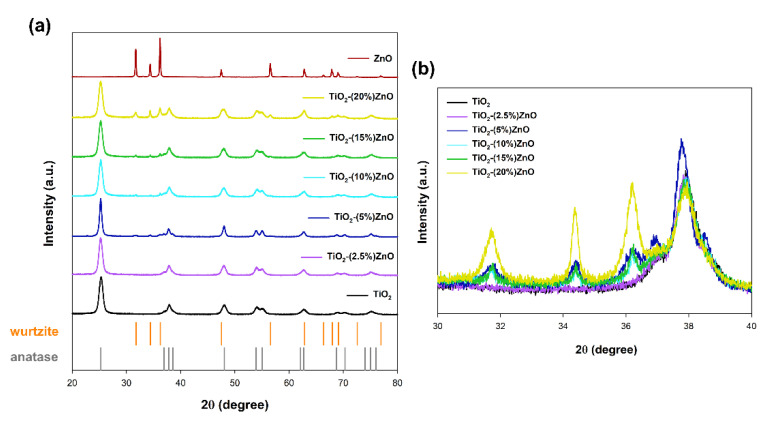
The XRD patterns (**a**) and focus on 2θ = 30–40° (**b**) for TiO_2_-ZnO systems and reference samples.

In the case of materials based on metal oxides, one of the most critical parameters is the crystal structure, which determines the subsequent application properties. The obtained XRD patterns are presented in Figure 4.

For both reference samples of titanium dioxide and zinc oxide, the crystal structure of tetragonal anatase (space group *I4_1_/amd*, no. 141; databased card no. 9009086) and hexagonal wurtzite (space group *P6_3_mc* no. 186; databased card no. 2300112) was recorded, respectively. Many reports show the anatase and wurtzite structures for samples synthesized with hydrothermal treatment [39]. Similar observations were also demonstrated in our earlier works on the one-step hydrothermal-assisted synthesis of TiO_2_-ZnO binary systems [40].

In the next stage, the obtained XRD patterns for TiO_2_-ZnO systems were analyzed. Based on the obtained results, it was shown that the addition of ZnO ≤ 5 wt.% caused the crystallization of the wurtzite structure, as confirmed by the diffraction bands at 2θ = 31.6, 34.3, as well as 36.1. On the other hand, only the anatase structure was observed for the material TiO_2_-(2.5%)ZnO. Additionally, no shift on the XRD pattern was noted for the above-mentioned two-component material compared to the reference titania sample (Figure 4b). Therefore, it should be supposed that the crystallization of ZnO can be inhibited by further growth of TiO_2_ in the next step of the hydrothermal treatment.

The average crystallite size and phase composition were determined in the next step of the crystallographic analysis (see Table 2).

By analyzing the presented crystallinity results, it was shown that the crystallite size of 10 nm characterized the reference titania material. In contrast, for zinc oxide, an average crystallite size of 45 nm was determined. According to the previously presented data, no crystalline phase of wurtzite was observed for the material containing 2.5 wt.% of ZnO (TiO_2_-(2.5%)ZnO). However, for the sample mentioned above, an increase of the crystallites size of anatase (the largest crystallite size of all analyzed materials) was confirmed. Considering the possible reason for the lack of ZnO crystallinity in the discussed material, it is necessary to quote Ludi and Niederberger’s [41] work. The authors indicate that the crystallization process can be inhibited if the amount of locking agent is high. Moreover, only single ZnO particles were observed for these materials. Additionally, Garcia and Samancik [42] found that many modification agents can determine the growth of ZnO crystal in different directions. Scientists have shown, among others, the addition of citrate ions inhibits axial growth. However, the increase in crystal width could imply that mentioned ions accelerate the equatorial growth of ZnO crystal. Likewise, the modification agents might suppress nucleation, allowing higher supersaturation in the solution, forming larger crystal dimensions overall. However, the modifying agent used in our work, TiO_2_ modified with triethylamine, due to amine groups, attaches the non-polar facets of the zincite crystal, thereby cutting off the access of Zn^2+^ ions to them, leaving only the polar (001) face for epitaxial growth [20,43]. For this reason, at a low concentration of Zn^2+^ ions, the formation of the ZnO structure may be inhibited. In the case of other synthesized TiO_2_-ZnO systems, the presence of both tetragonal anatase and hexagonal wurtzite phases was noted—in a similar ratio as assumed. On the other hand, when analyzing the values of the average size of crystallites, attention should be paid to the significant size of ZnO crystallites in the range of 43–45 nm, which may also confirm the previous considerations wurtzite crystallization process. It was noted that the TiO_2_-based systems containing from 2.5–10 wt.% of ZnO were characterized by a larger size of anatase crystallites, which is related to their growth during the secondary hydrothermal treatment.

**Figure 5:**
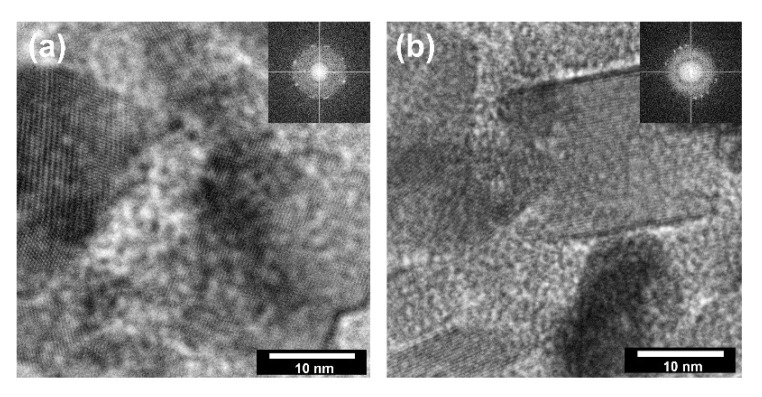
The HR-TEM and FFT images for: (**a**) TiO_2_-(2.5%)ZnO; (**b**) TiO_2_-(20%)ZnO.

In the next stage of research on the crystal structure, determining the direction of crystal growth and defining the main planes influencing their growth was evaluated. Thus, the HR-TEM analysis (Figure 5) was carried out, considering the growth of metal oxide crystals during hydrothermal treatment.

According to the available scientific literature, the orientation of the growth of crystalline zinc oxide under certain heat treatment conditions is well defined [44]. Therefore, HR-TEM imaging focused mainly on TiO_2_ particles for which the synthesis conditions have a determining the influence on the crystal growth. In HR-TEM and FFT images (Figure 5), TiO_2_ particles in TiO_2_-ZnO systems are correctly oriented with respect to the impinging electron beam showing lattice fringes 0.23 nm apart (d_004_ = 0.2378 nm) [25]. Thus, they correspond to {001} surfaces. Based on the presented HR-TEM data, as well as the available scientific literature, including Mino et al. [45], it was confirmed that the planes (101), (004), and (200) had a determining influence on the formation of anatase nanocrystals. Therefore, the crystallite size was presented in the next step for the planes mentioned above (Table 3).

Based on the data presented in Table 3, it can be assumed, above all, that the crystallite sizes for the crucial crystalline planes are similar to each other. Additionally, it was noted that the crystallites of planes (004) and (200) slightly decreased with increasing zinc oxide addition, which may indicate the influence of ZnO on the anatase crystal structure.

Based on the XRD results obtained, it was shown that the proposed two-stage synthesis methodology based on the modification of titania and then the synthesis of TiO_2_-ZnO systems resulted in preparation materials with two crystalline phases—tetragonal anatase and hexagonal wurtzite. In addition, it was noted that no crystalline phase of wurtzite was observed in the case of materials containing a small addition of ZnO (≥5%). This can be caused by the high amount of capping agent (in this case, nanocrystalline titanium dioxide), leading to the formation of single ZnO particles. Furthermore, attention should be paid to the refined lattice parameters for TiO_2_-ZnO systems that are in good agreement with other literature reports [46,47].

#### 3.2.2. Morphology and Surface Composition

**Figure 6:**
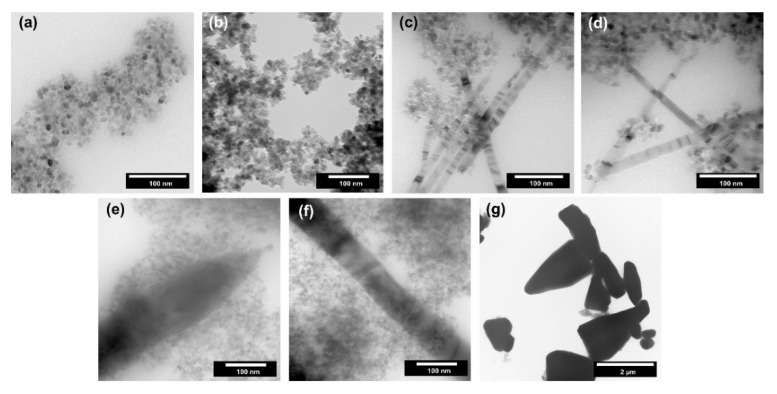
The TEM images for TiO_2_-ZnO systems and reference samples: (**a**) TiO_2_; (**b**) TiO_2_-(2.5%)ZnO; (**c**) TiO_2_-(5%)ZnO; (**d**) TiO_2_-(10%)ZnO; (**e**) TiO_2_-(15%)ZnO; (**f**) TiO_2_-(20%)ZnO and (**f**) ZnO.

The shape and size of nanoparticles can determine their application properties, e.g., defective structure or different access to individual planes. Therefore, a detailed analysis of morphology and microstructure is one of the critical parameters in materials science. For the obtained TiO_2_-ZnO systems, transmission electron microscopy was performed, and the results are presented in Figure 6.

Single spherical and cubic nanoparticles with a size of about 10 nm were observed for the reference titanium dioxide sample (Figure 5a). Based on the above-described crystallinity results, the average size of crystallites for titania was determined to be 10 nm, which is a similar value to the size of a single particle. Thus, it was proved that TiO_2_ was in the form of single nanocrystalline particles. For the sample containing 2.5 wt.% of ZnO (TiO_2_-(2.5%) ZnO) (Figure 5b), only the single nanoparticles (about 10 nm in size) were noted, which is similar to the morphology observed for the reference titania material. The increase of ZnO addition to 5–10 wt.% (TiO_2_-(5%)ZnO and TiO_2_-(10%)ZnO) (Figure 5c,d) resulted in the presence of a new morphological structure. ZnO nanowires with a 10–20 nm diameter were observed for the materials mentioned above, and the structure of nanocrystalline TiO_2_ particles was also found. A further increase in ZnO content (TiO_2_-(15%)ZnO and TiO_2_-(20%)ZnO) (Figure 5e,f) resulted in an increase in the diameter of ZnO nanowires (diameter about 50 nm). The change of ZnO addition did not affect the morphology of the TiO_2_ phase. On the other hand, for the ZnO reference sample, pyramidal and rod structures were approximately 2 µm (Figure 5g). The structures mentioned above are described in the scientific literature for ZnO materials synthesized in the presence of amines in a basic medium [20,43]. It should be noted that both pyramidal and rod-like structures were not observed for the TiO_2_-ZnO systems which only ZnO nanowires were found. The main reason for the formation of ZnO nanowires is the use of TiO_2_ modified with triethylamine.

**Figure 7:**
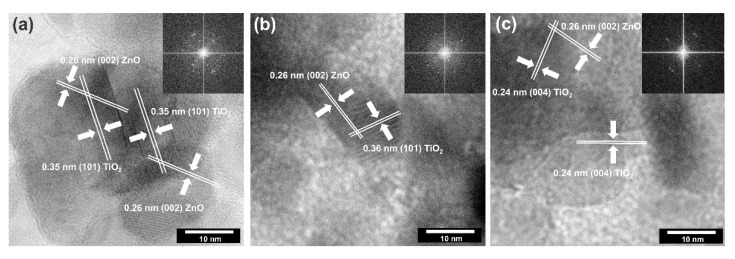
The HRTEM and FFT images for: (**a**) TiO_2_-(5%)ZnO; (**b**) TiO_2_-(10%)ZnO; (**c**) TiO_2_-(15%)ZnO.

The high-resolution transmission electron microscopy with fast Fourier transformation was carried out (Figure 7) to confirm the existence of a surface junction between titanium dioxide and zinc oxide.

Based on the presented HRTEM and FFT images, the high crystallinity of the analyzed systems was confirmed due to observed crystallographic spacing. It was found that selected TiO_2_-ZnO samples have spacing characteristics for a plane (101) and (004) of anatase [43] and (002) of wurtzite [48]. Attention was also paid to the crossing of lattice fringes of titania and zinc oxide. Such connections have been described as a heterojunction which is widely known in the scientific literature [31,49].

**Figure 8:**
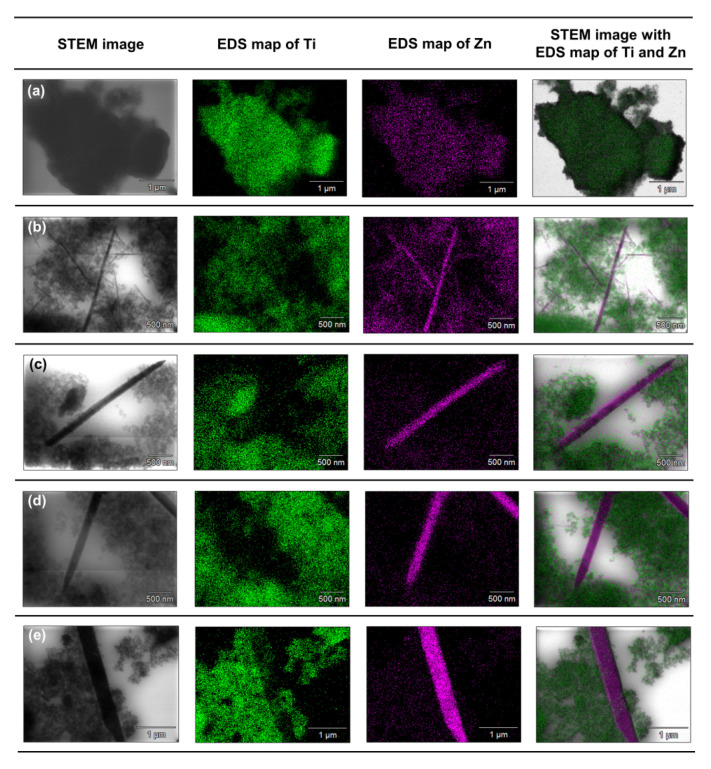
The results of EDX mapping for: (**a**) TiO_2_-(2.5%)ZnO, (**b**) TiO_2_-(5%)ZnO, (**c**) TiO_2_-(10%)ZnO, (**d**) TiO_2_-(15%)ZnO, (**e**) TiO_2_-(20%)ZnO systems.

To accurately determine the position of individual components in the synthesized systems, an analysis of the EDX mapping (Figure 8) and EDXRF (Table 4) was performed.

Based on the presented EDX data, it can be concluded that for the TiO_2_-(2.5%)ZnO sample, aggregates of titania particles were observed, while ZnO is present only on single nanoparticles. The described observations confirm that with a low concentration of ZnO additive, titanium oxide can play a role as a capping agent, which inhibits ZnO crystallization and precludes obtaining morphological structures known from the literature [5,50]. In the case of the TiO_2_-(5%)ZnO sample, apart from TiO_2_ nanoparticle aggregates, there are also single particles and ZnO nanowires of various lengths. The presence of both nanowires and individual ZnO nanoparticles indicates that the increase in ZnO addition to 5 wt.% results in the crystallization of wurtzite and the formation of crystalline nanowires. However, some of the particles are still blocked by titanium dioxide, which is consistent with the XRD data obtained for the material mentioned above. The content of the crystalline phase of wurtzite (3.8%) was determined as expected. For the remaining analyzed materials (ZnO = 10, 15, and 20 wt.%) apart from the phase of nanocrystalline TiO_2_ particles, the structure of ZnO nanowires without the presence of single particles was also noted, which may indicate that with the ZnO content equal to 10 wt.%, titanium dioxide ceases to be an inhibition of crystallization growth of ZnO. In addition, based on the results of EDS mapping (Figure 8), it was indicated that TiO_2_ nanoparticle aggregates are observed near crystalline ZnO nanowires. Such a result may also confirm the proposed nanofiber growth mechanism based on interactions of the modified titania sample with the non-polar facets of the zincite crystal.

On the other hand, attention should be paid to the results of the EDXRF analysis (Table 4). It was found that the determined percentages of titanium dioxide and zinc oxide are similar to the theoretical values assumed during synthesis. Therefore, it seems that the observed single ZnO particles in TiO_2_ nanocrystalline aggregates are not incorporated in the titanium structure, but are amorphous ZnO particles whose crystal growth has been inhibited. Additional confirmation of the absence of ZnO particles incorporation was also showed by XRD data (crystal lattice parameters), which were close to the literature values [51,52].

There are many reports in the available scientific literature about the connections between titanium oxide and zinc oxide. Among others, Siwińska-Stefańska et al. [53] and Perez-Gonzalez et al. [54] confirmed that zinc oxide could inhibit the growth of zinc oxide during one-step synthesis anatase structure. For this reason, the advantageous solution seems to be the synthesis in two stages carry out, as presented, among others, by Sartori et al. [55] and Cheng et al. [56], who used commercial titanium dioxide (P25) for synthesis. However, the use of P25 is also associated with certain disadvantages related to its homogeneity, which was pointed out by Ohtani [57]. At the same time, the homogeneity of the material is one of the crucial parameters that influence physicochemical properties. For this reason, it seems advantageous not only to synthesize a homogeneous and comprehensively characterized titanium dioxide, but also to modify it to obtain the designed oxide systems. The *in-situ* modification method used in our work made it possible to influence the next stage of synthesis and obtain the determined crystalline ZnO nanowires.

#### 3.2.3. Optical Properties

**Figure 9:**
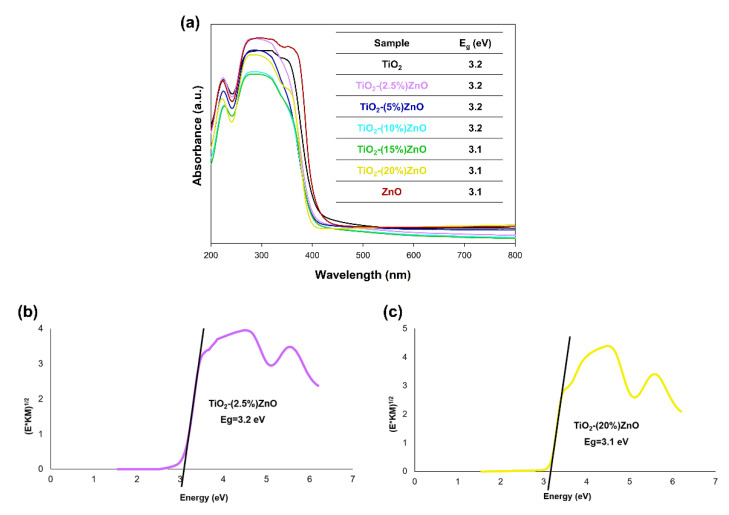
The absorption spectra of TiO_2_-ZnO systems and reference samples (**a**) and Kubelka−Munk function (E*KM)^1/2^ as a function of the photon energy of (**b**) TiO_2_-(2.5%)ZnO and (**c**) TiO_2_-(20%)ZnO samples.

Due to the semiconductor properties of both components included in the oxide systems, a study of diffuse reflectance spectroscopy (Figure 9) was carried out.

Based on the results of the DRS/UV-Vis analysis (Figure 9a), it was shown that all analyzed materials had one wide absorption band in the 400–200 nm wavelength range, which proves the absorption of UV radiation. Next, the energy band gap of the TiO_2_-ZnO systems and reference samples were calculated by the Kubelka–Munk theory. In the case of the graphs of the Kubelka–Munk function as a function of energy, it was found that the band gap energy for the reference samples TiO_2_ and ZnO were 3.2 and 3.1 eV, respectively. The presented photon energy (E_g_) results for titanium dioxide and zinc oxide are consistent with scientific knowledge [58]. On the other hand, the band gap energy results from 3.1 to 3.2 eV were obtained for the obtained two-component systems. The values mentioned above are similar to the results obtained for the reference samples—TiO_2_ and ZnO. Furthermore, attention should be paid to the lack of shifts in the value of the bandgap energy results for the TiO_2_-ZnO systems, which additionally confirm that the obtained materials contain only the two phases—anatase and wurtzite without the presence of mixed crystal structures, e.g., ZnTiO_3_ as well as Zn_2_TiO_4_.

#### 3.2.4. Characteristics of Functional Groups

**Figure 10:**
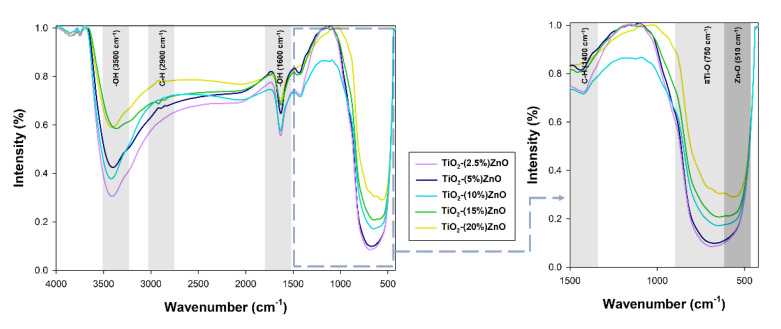
The FTIR spectra for TiO_2_-ZnO systems.

The FTIR (Figure 10) analysis was performed to confirm the effectiveness of the proposed synthesis methodology.

On the presented FTIR spectra, bands corresponding to vibrations: stretching -Ti≡O (750 cm^−1^), stretching Zn-O (510 cm^−1^), and bending and stretching C-H (1400 cm^−1^ and 2900 cm^−1^) were observed. Moreover, a band originating from stretching vibrations of the hydroxyl group (3500 cm^−1^), as well as a band from physically adsorbed water (1600 cm^−1^), were noted [9]. The bands derived from the C-H groups are related to the organic modifier used in the research—triethylamine [32].

On the spectra of the analyzed oxide systems, bands characteristic of both titania (-Ti≡O) and zinc oxide (Zn-O) were observed. Only for systems containing 2.5% and 5% wt. ZnO, no zinc oxide bands were noted, which may be explained by their coverage by broadband of titanium dioxide. Nevertheless, the presented results of the FTIR analysis prove the correctness of the proposed methodology for the synthesis of TiO_2_-ZnO oxide systems using the hydrothermal-assisted method.

#### 3.2.5. Thermal Stability

**Figure 11:**
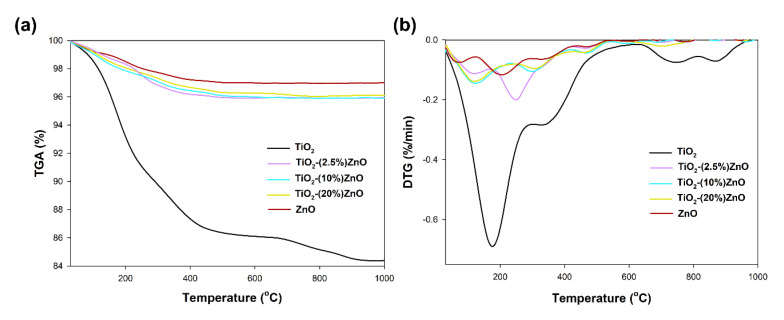
TGA (**a**) and DTG (**b**) curves of TiO_2_-ZnO systems and reference samples.

In the last stage of evaluating the physicochemical properties, the thermal stability of the selected synthesized materials was determined. Figure 11 shows the obtained TGA/DTG curves.

For the reference titanium dioxide sample, a total mass loss of 15% was recorded. For this sample, the TGA/DTG profile exhibits five features. The first broad peak can be ascribed to the evaporation of physically adsorbed and the removal of chemically bonded water and occurred in the range of temperature 125–225 °C. The presence of physically bound water was also found by FTIR analysis (Figure 10). Next, the peak visible at temperatures 350 °C can be ascribed to the decomposition of residual organic modifier—triethylamine [59]. Finally, the two small peaks at temperature 750 °C and 850 °C can be associated with a change in the titanium dioxide crystal structure—phase transition and crystal growth [60]. On the other hand, for the second reference sample (ZnO), a weight loss of 3% related to the evaporation of physically adsorbed water (117 °C) as well as the removal of chemically bonded water (205 °C). For TiO_2_-ZnO systems, a similar weight loss (about 4%) is observed for all analyzed materials. For the material containing 2.5 wt.% of ZnO, two mass losses were noted—the first related to the removal of adsorbed water (121 °C) and the second in the range of 250–300 °C, resulting from the removal of physically bounded water, as well as the decomposition of organic parts—triethylamine and acetate groups. For the TiO_2_-(10%)ZnO and TiO_2_-(20%)ZnO samples, only two peaks on the DTG curves were recorded (125 °C), related to water evaporation and residual organic matter (305 °C), respectively. For TiO_2_-ZnO materials, no peaks at 750–850 °C were observed in the DTG plot, which may indicate that the addition of ZnO stops the phase transformation as well as the growth of anatase crystals. The inhibition of the growth of the crystalline anatase phase was observed on the basis of the XRD data, where despite the re-treatment, the anatase crystallites were similar to the reference material. Based on the obtained results, it was shown that TiO_2_-ZnO oxide systems are characterized by high thermal stability. It was additionally observed that even a small addition of ZnO (2.5 wt.%) improved the thermal stability of titania.

#### 3.2.6. Photocatalytic Activity

**Figure 12:**
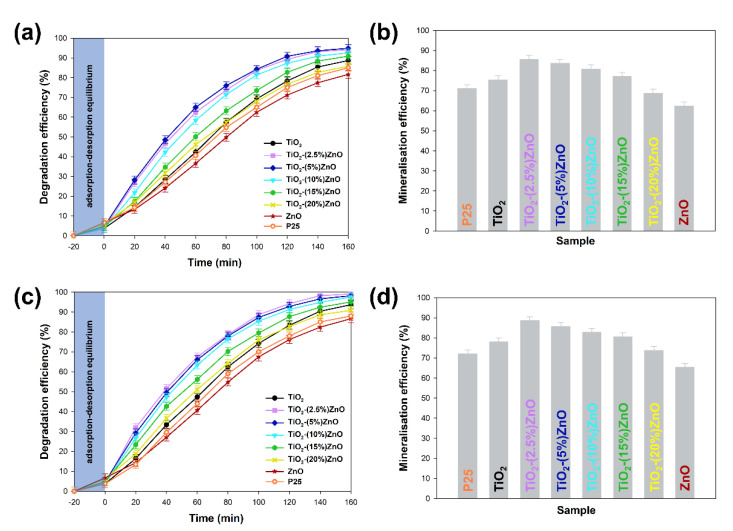
The results of degradation and mineralization of 4-chlorophenol with used 10W (**a**,**b**) and 20W (**c**,**d**) UV-LED light source.

The obtained two-component systems were tested as active photocatalysts in the degradation process of 4-chlorophenol, bearing in mind the widely described photocatalytic properties of both titanium dioxide and zinc oxide. The critical element of the conducted photo-oxidation tests was applying low-power UV-LED lamps as a light source. Moreover, the influence of lamp power on the degradation process of the tested organic pollutant was determined. The obtained degradation curves, as well as the mineralization efficiency, are summarised in Figure 12.

At the outset, it should be noted that regardless of the power of the light source, the analyzed materials were characterized with high photoactivity. By applying a 20 W lamp, about 3–5% higher removal efficiency was observed than when 10 W light source was used. Based on the conducted photo-oxidation tests, it was shown that the TiO_2_ and ZnO reference samples were characterized by correspondingly the degradation efficiency—88 and 81%, as well as mineralization—75% and 62% of the tested impurity. In case the two-component materials containing from 2.5 wt.% to 15 wt.% of ZnO, an increase in photocatalytic activity (both in the case of degradation and mineralization) was noted in relation to the reference titanium dioxide sample. For TiO_2_-(2.5%)ZnO, TiO_2_-(5%)ZnO, and TiO_2_-(10%)ZnO photocatalysts, a similar photodegradation curve was recorded, which may be related to the similar value of the band gap energy of the above materials. The mentioned materials showed degradation of 4-chlorophenol equal to about 94%, while the mineralization efficiency was in the range of 85–80%. Increasing the ZnO addition to 15 wt.% (TiO_2_-(15%)ZnO sample) resulted in a slight deterioration in the degradation efficiency (91%) of 4-chlorophenol as compared to the materials described above. For the TiO_2_-(20%)ZnO sample, a lower degradation (85%) and mineralization (68%) yield of the organic pollutant among the tested TiO_2_-ZnO systems were noted. The obtained results of photo-oxidation tests were compared with the commercially available photocatalyst—P25. The observed degradation efficiency reached 88%, while the mineralization efficiency was 72% regardless of the light source used. This allows indicating that the obtained TiO_2_-ZnO systems are characterized by a higher degree of removal of the tested pollutant, which may be related to a different photocatalytic mechanism and different absorption of radiation emitted by UV-LED diodes.

**Figure 13:**
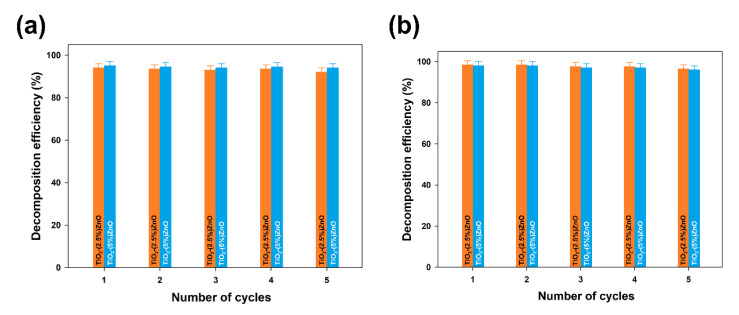
The efficiency of 4-chlorophenol decomposition in the presence of TiO_2_-(2.5%)ZnO and TiO_2_-(5%)ZnO samples during five successive cycles using 10W (**a**) and 20W (**b**) UV-LED light source.

Furthermore, the TiO_2_-ZnO systems with the highest removal efficiency (TiO_2_-(2.5%)ZnO and TiO_2_-(5%)ZnO samples) were selected for reusability studies. Five successive cycles were carried out to evaluate the effectiveness of the photocatalysts after their recovery. The data are shown in Figure 13.

At the end of the first run of photo-oxidation tests, the TiO_2_-ZnO samples were separated from the aqueous solution by filtration. The separated materials were then reused without any purification. In each case, the efficiency of 4-chlorophenol removal using TiO_2_-ZnO systems was above 90%, even after 5 catalytic cycles, regardless of the UV-LED light source used. Such a result confirms that the synthesized systems can be used many times in photo-oxidation processes.

Based on the obtained results of the photo-oxidation test, it was found that the high removal of 4-chlorophenol characterizes the obtained TiO_2_-ZnO systems. However, analyzing the obtained results, attention should be paid to the current standards of 4-chlorophenol concentration in the environment. According to the World Health Organization, the presence of chlorophenols in drinking water is equal to 2 µg/dm^3^ [61]. Hence, the obtained in the presented study mineralization efficiencies lead to the significant reduction of the concentration of 4-chlorophenol content in the aquatic environment and the fulfillment of the current WHO standards regarding the chlorophenols content in drinking water.

One of the fundamental observations resulting from the performed photo-oxidation tests was the influence of ZnO addition on the obtained photodegradation efficiency. It is well known that zinc oxide is a frequently used “second oxide” in combination with titanium dioxide, thereby improving the photocatalytic performance of the resulting binary oxide system. For example, Upadhyay et al. [62] indicated that TiO_2_-ZnO material is one of the most popular semiconductors couplings. However, the synthesized (molar/mass ratio) composition is one of the most important parameters, because it directly impacts the crystal structure and morphology. Xu et al. [63] pointed out that the improvement of photocatalytic properties compared to pure TiO_2_ can occur at the atomic ratio Ti/Zn = 3/1. Additionally, Fu et al. [64] found that the photo-oxidation activity increases with increasing the TiO_2_ doping content until the TiO_2_-ZnO ratio is more than 5% ZnO, which can explain the observed increase in photodegradation efficiency for TiO_2_-(2.5%)ZnO and TiO_2_-(5%)ZnO materials. On the other hand, the high photoactivity of the TiO_2_-(10%)ZnO system can be explained by using a light source with a specific wavelength and the value of the energy band gap similar to the previously described materials. Moreover, for the rest, materials containing 10 wt.% ZnO can be a highly efficient photocatalyst, which was highlighted by Siwińska-Stefańska et al. [40]. The decrease of the photocatalytic activity in samples containing 15% and 20% ZnO results, among others, from the increase of carriers charge recombination effect [65]. Furthermore, the higher zinc oxide content can cause an increase in opacity and light scattering, adversely influencing photon absorption. Based on the obtained results, it was shown that the addition of ZnO ranging from 2.5 wt.% to 10 wt.% of ZnO significantly improves the photocatalytic degradation of 4-chlorophenol in comparison to the reference TiO_2_ sample. Additionally, it was observed that to improve the photocatalytic activity of the systems, ZnO does not have to be in a crystalline form—wurtzite. It seems that ZnO nanoparticles dispersed in TiO_2_ aggregates can also effectively improve the photocatalytic performance in UV-LED light.

Another important conclusion is the lack of a decisive influence of the power of the used light source on the obtained degradation efficiency of the tested impurity. In our research, photocatalytic LED reactors based on strips of light-emitting diodes were used. Despite using SMD diodes, the discussed technological solution made it possible to use a uniform beam of light supplied to the entire reactor volume, which resulted in high degradation efficiency. To the best of our knowledge, in the available scientific literature, work concerning the use of LEDs in photocatalytic processes focuses on the use of COB modules [66] or LED boards [17,18]. The main problem with the solutions mentioned above is the limited light flux, which decreases with the distance of the LED light source from the photo-reaction system, as described in detail by Casado et al. [17]. For this reason, the solution presented by us seems to be interesting, which allows for even illumination of the reaction system using a low power of the light source. An additional advantage is using SMD LEDs, which are commonly used in commercially produced lighting fixtures despite their inferior parameters to COB systems. Therefore, they seem to be a more beneficial and cheaper solution at the current stage of LED technology development. However, the technological solution used should also consider the lack of visible influence of the power of the light source on the achieved degradation efficiency. According to the assumptions, the LED strip generates about 10 W per meter of the strip (60 LEDs). Therefore, in the case of a lamp with a higher power, it was necessary to use 120 LEDs to obtain complex radiation power. On the other hand, an increase in the number of diodes is also associated with an increase in light scattering and its partial reflection. Additionally, the light source used in our experiment had a specific wavelength of 395 nm. Consequently, to achieve maximum radiation absorption, it was possible to use a lamp with lower power. Nevertheless, it seems that the further development of photocatalysis based on LED light sources is another step in integrating photodegradation processes into environmental protection strategy.

The next step in evaluating the photocatalytic properties of the synthesized two-component oxide systems was to propose a possible photodegradation mechanism. Due to the location of the conduction (CB) and valence bands (VB) of the semiconductors included in the described oxide systems, titanium dioxide and zinc oxide, the probable mechanism of the photocatalytic reaction is the heterojunction type II [67]. The photo-excited electrons are transferred from the ZnO conduction band to CB TiO_2_. This transfer can occur directly between the semiconductors due to the favorable energetics of the conduction bands’ position [68]. The holes are transferred simultaneously from the titanium dioxide VB to the zinc oxide VB, reducing the likelihood of recombination and increasing the lifetime of the charge carriers. The oxidation process occurs in the zinc oxide valence band by reacting the holes with adsorbed water or surface hydroxyl groups, resulting in the formation of hydroxyl radicals. The reduction process occurs in the conductivity band of titanium dioxide, where radicals of superoxide anions are formed, which can then be transformed into active oxygen species. Based on the scientific knowledge, Stafford et al. [69] found that the critical parameter influencing the photooxidative of 4-chlorophenol was the presence of the hydroxyl radical (*OH), which is described as the primary oxidant in the impurity mentioned above. Furthermore, the scientists indicated that holes (h^+^) were responsible for the degradation process to a lesser extent [70]. On the other hand, Li et al. [71] showed that the major degradation road for 4-chlorophenol is the hydroquinone pathway. Hydroquinone is mainly oxidized to 1,2,4-benzenetriol, and most of the ring cleavage comes from this compound. However, a modest amount of tetraol and two additional acyclic six-carbon compounds can also be observed. The authors also indicated that the presented photo-oxidation pathway is suchlike to other aromatic compounds, including quinoline, naphthalene, and pyridine. On the other hand, after opening the ring, further decomposition into shorter organic compounds occurs, and consequently their mineralization. Finally, the main reactions in the photocatalytic removal of 4-chlorophenol are proposed as follows hydroxylation, dihydroxylation, hydrations, and decarboxylation.

**Figure 14:**
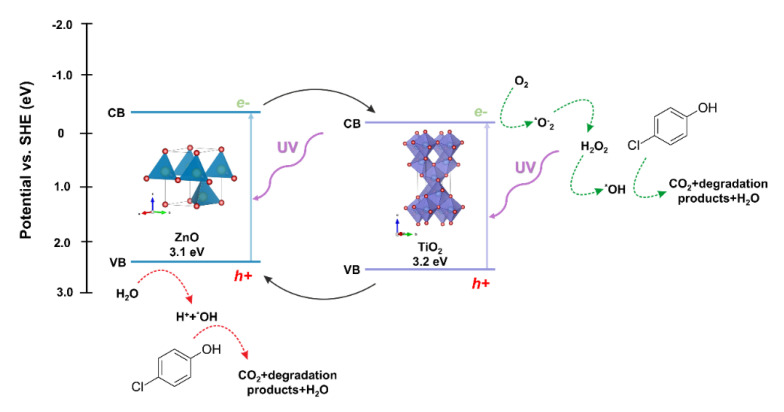
The proposed mechanism of photo-oxidation for 4-chlorophenol using TiO_2_-ZnO systems as photocatalysts.

The proposed mechanism of degradation of the model dye solution in the presence of the TiO_2_-ZnO oxide system is shown in Figure 14.

Phenol and its derivatives, such as chloro- and nitrophenols or bis-phenols, are recognized by the World Health Organization (WHO) as significant impurities posing a threat to aquatic organisms [72]. For this reason, many research teams are working on the degradation of the pollutants mentioned above. An overview of the available scientific knowledge on the photocatalytic degradation of 4-chlorophenol is summarized in Table 5.

According to the presented review of the available scientific literature on the photodegradation of 4-chlorophenol, various photoactive materials are used, including oxides such as TiO_2_, ZrO_2_, ZnO, and SnO_2_. The results of photodegradation efficiency obtained by us were better or similar to those presented in the literature. However, the main difference is the UV light source used. According to the presented data, scientists used high-power mercury or xenon lamps (125–500 W) in most of the mentioned works. Apart from xenon and mercury lamps’ high operating costs, they are also characterized by having a short lifetime of 10,000 h and 500–1500 h, respectively [78]. On the other hand, for the LEDs, the lifetime can reach 100,000 h [79], where the minimum lighting time is 30,000 h [18]. An additional problem with the use of high-power mercury or xenon lamps is the high energy loss in the form of heat. At the same time, in LED lamps, some electricity is converted into heat, but much less, and the cooling system is limited to a passive heat sink. Finally, LEDs are primarily widespread technology what makes them cheaper and less energy-consuming than other solutions such as Hg or Xe lamps [78], and an additional advantage of LEDs is the possibility of choosing an LED well-matched to the spectrum of the target photocatalyst. For this reason, obtaining high photodegradation efficiency with the use of a low-power UV-LED lamp shows that in the case of a properly selected photocatalyst (in our case, TiO_2_-ZnO systems), LEDs can be an alternative to conventional UV light sources, and most importantly, with due to their advantages such as long life time, low energy consumption as well as high light efficiency, they are environmentally friendly.

Based on the obtained results, it was confirmed that using a UV-LED lamp and the fast and efficient hydrothermal synthesis of TiO_2_-ZnO systems. It is possible to obtain particles that exhibited enhanced photocatalytic performance. Thus, these promising TiO_2_-ZnO photocatalysts may also be applied in the removal of other organic impurities.

#### 3.2.7. Photovoltaic Properties

**Figure 15:**
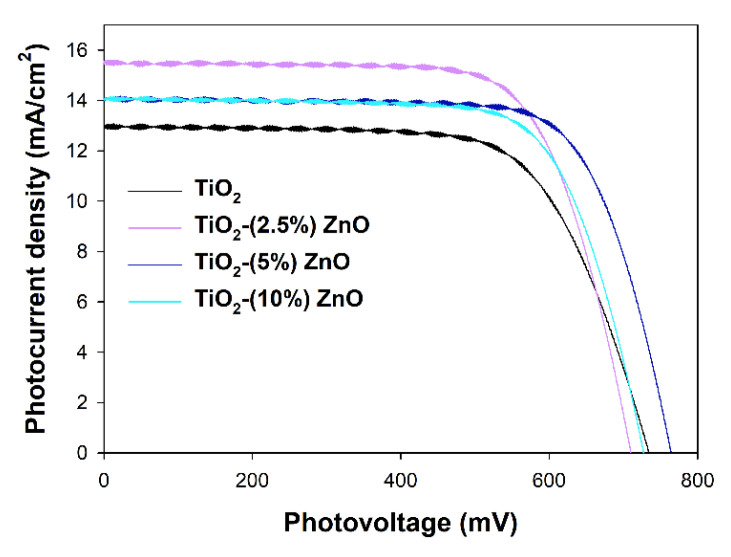
*I–V* curves registered for investigated TiO_2_-ZnO cells.

The most active ZnO containing photocatalysts, i.e., TiO_2_-(2.5%)ZnO, TiO_2_-(5%)ZnO, and TiO_2_-(10%)ZnO, and reference TiO_2_ material have been selected to investigate their properties as the semiconducting layer of the working electrodes in DSSCs. Assembled DSSCs have been characterized with *I–V* curves presented in Figure 15, and the parameters of the device red from the *I–V* curves have been collected in Table 6. The overall photon-to-current efficiencies (*η*) of all DSSCs utilizing ZnO-containing materials have been improved compared to TiO_2_ cells. The *η* increase is the direct result of the increase in two other parameters, i.e., fill factors (*FF*), which is the energy loss related to inherent resistance in the photovoltaic cells [80], and photocurrent densities (*J_SC_*). The growth in *FF* values may suggest that the electron transport abilities of the ZnO-containing materials have been increased and/or the recombination rate has been suppressed. Both effects mentioned above also affect the *J_SC_* values, but additionally, *J_SC_* depends on the number of dye molecules adsorbed on the working electrode surface. The amounts of the adsorbed dye molecules on the investigated working electrodes have been determined, and the results have been collected in Table 7 (see *N_dye_* value). Surprisingly, the higher the ZnO content in the semiconducting material, the lower the adsorbed dye amount. The decrease in the *N_dye_* amount simultaneously with the improvement of the *J_SC_* registered for ZnO-containing cells suggests that the recombination rate and electron transport abilities effects have prime importance in the efficiencies of investigated cells.

The third value that influences *η* is the open circuit photovoltage (*V_OC_*), mainly depending on the energy level difference between the Fermi level of the semiconductor and the present in the electrolyte solution redox mediator’s Nernst potential [81]. The *V_OC_* values are pretty random in the presented devices, and no visible tendency may be observed. As mentioned above, the band gaps of investigated materials selected for the photovoltaic experiments do not differ, and all are equal to 3.2 eV. As in all investigated DSSCs, the same electrolyte solution has been used, and nothing suggests the shift of the Fermi levels of the utilized semiconductors. The observed situation makes some difficulties in interpreting the obtained results and leads to a significant decrease of the *η* value of TiO_2_-(2.5%)ZnO cell even if it is characterized with still high *FF* and the highest *J_SC_* values.

**Figure 16:**
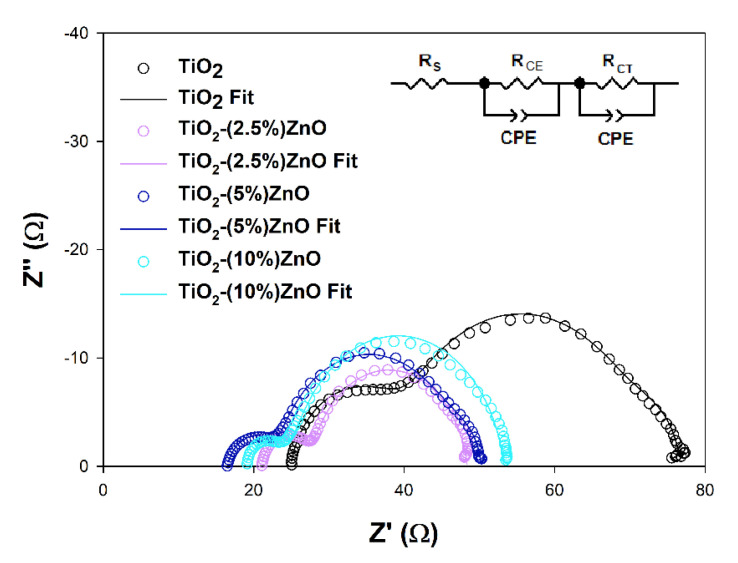
Nyquist plots of the EIS results registered for investigated cells and the scheme of equivalent circuits used for the results fitting.

Electrochemical impedance spectroscopy (EIS) is a valuable tool to explain deeper the reasons for some DSSCs’ behaviors. The EIS results obtained for investigated cells are presented in Figure 16 and summarized in Table 7.

The *R_S_* and *R_CE_* values, which are the series resistance, mainly depend on the parameters of the measuring system used and the resistance of the counter electrode used to build the cells, respectively. As it can be seen, they do not differ significantly between cells tested, do not depend on the semiconductor materials used, and do not considerably impact differences in the efficiency of the devices discussed [80]. The effect may cause the increase in the *R_CE_* parameter observed for the TiO_2_ cells resulted from the increase of the *R_CT_* parameter in these cells or the minor differences in the Pt film thickness or discontinuities in the Pt layer [82,83]. More interesting for this study is the *R_CT_* value, which may be understood as the semiconductor/dye/electrolyte interface resistance. In general, the higher the registered *R_CT_* value, the lower the *J_SC_* of the cell, except for the 5 and 10% ZnO cells pair, where the *J_SC_* values are equal to each other, and the *R_CT_* values are different. The above observation suggests that additional effect has to be involved in the photovoltaic process of investigated cells. The electron lifetimes (*τ*) have been estimated from the highest frequency point (*f*) of the *R_CT_* semicircle using equation *τ* = (2*πf*)^−1^ to get a deeper insight into electron processes occurring in the cells presented. It is well known that the electron lifetime depends on the semiconducting material’s trapping abilities, but intense trapping disturbing the electron transport and may cause a decrease in cell efficiency [81,84]. The fact that the electron trapping process is too effective may be why the higher *R_CT_* value has been observed in TiO_2_-(10%)ZnO cells. The observed *FF* values may also support the above interpretation. Some authors state that the electron lifetime may also affect the *V_OC_* value of the DSSC by the relaxation of the injected electrons trapped into the semiconductor [85]. Based on the above literature findings, one may say that the slightly higher *τ* value estimated for TiO_2_-(2.5%)ZnO cells may lead to *V_OC_* loss. However, the intense electron trapping in the TiO_2_-(10%)ZnO material may suppress the relaxation and support relatively high VOC keeping.

## 4. Conclusions

One of the main goals of the presented work was to obtain two-component TiO_2_-ZnO systems using a simple two-stage hydrothermal route. Based on the XRD results obtained, it was shown that the proposed two-stage synthesis methodology resulted in obtaining materials with two crystalline phases—anatase and wurtzite. Additionally, it should be noted that no crystalline phase of wurtzite was observed for materials containing a small addition of ZnO (≥5 wt.%). The lack of the wurtzite crystalline phase led to the formation of single ZnO particles marked on the EDX maps.

The important objective of this research was to analyze the photocatalytic properties of the synthesized TiO_2_-ZnO systems in the UV-LED light range. It was proved that the TiO_2_-ZnO materials (especially TiO_2_-(2.5%)ZnO and TiO_2_-(5%)ZnO samples) exhibit high photo-oxidation efficiency in the removal of 4-chlorophenol. It seems that ZnO nanoparticles dispersed in TiO_2_ aggregates were the main factor causing the improvement in the photocatalytic performance in UV-LED light. The presented materials have also exhibited good properties when used as semiconducting layers in DSSCs. The best photovoltaic parameters have been found in the case of the TiO_2_-(2.5%)ZnO material, which was characterized by the optimal dye loading on the electrode surface as well as the lowest internal resistance.

## Figures and Tables

**Table 1:** Results of elemental analysis for modified titanium dioxide.

Sample	N (%)	C (%)	H (%)	S (%)
TiO_2_	1.8	3.0	2.0	0.0

**Table 2:** The average crystallite size and phase composition of the obtained TiO_2_-ZnO systems and references samples.

Sample	The Crystallite Size (nm)		Phase Composition (%)		Lattice Parameters			
	Anatase	Wurtzite	Anatase	Wurtzite	Anatase		Wurtzite	
					a (Å)	c (Å)	a (Å)	c (Å)
TiO_2_	10	–	100	–	3.78581	9.48861	–	–
TiO_2_-(2.5%)ZnO	11	–	100	–	3.78642	9.48820	–	–
TiO_2_-(5%)ZnO	11	43	97	3	3.91698	9.51081	3.24591	5.20956
TiO_2_-(10%)ZnO	10	43	90	10	3.78650	9.50409	3.25166	5.21054
TiO_2_-(15%)ZnO	10	44	85	15	3.78209	9.51045	3.25193	5.21110
TiO_2_-(20%)ZnO	10	44	81	19	3.78755	9.48887	3.25560	5.21411
ZnO	–	45	–	100	–	–	3.25608	5.21691

**Table 3:** The average dimension of crystal domains obtained by Scherrer analysis of the 100, 004, and 200 XRD peaks.

Sample	Crystallite Plane		
	(101)	(004)	(200)
	The Crystallite Size (nm)		
TiO_2_-(2.5%)ZnO	10	12	9
TiO_2_-(5%)ZnO	10	11	10
TiO_2_-(10%)ZnO	10	10	9
TiO_2_-(15%)ZnO	10	9	9
TiO_2_-(20%)ZnO	10	8	8

**Table 4:** The results of EDXRF analysis for TiO_2_-ZnO systems.

Sample	TiO_2_ (wt.%)	ZnO (wt.%)
TiO_2_-(2.5%)ZnO	97.3	2.7
TiO_2_-(5%)ZnO	94.9	5.1
TiO_2_-(10%)ZnO	89.9	10.1
TiO_2_-(15%)ZnO	84.9	15.1
TiO_2_-(20%)ZnO	80.2	19.8

**Table 5:** Comparison of the photocatalytic performance of ZnO particles.

Material	Degradation Conditions				Degradation Efficiency	Ref.
	Concentration	Amount of Photocatalyst	Type of Light Source	Power of Light Source		
TiO_2_-(2.5%)ZnO TiO_2_-(5%)ZnO	20 mg/dm^3^	0.1 g/dm^3^	UV-LED (395 nm)	10 W	95% (in 2.5 h)	This work
ZnO-rGO	5 mg/dm^3^	0.1 g/dm^3^	Hg–Xe lamp	200 W	94% (in 1 h)	[73]
P- modified TiO_2_	200 µmol/dm^3^	0.1 g/dm^3^	Hg lamp	11 W	98% (in 4 h)	[74]
MFe_2_O_4_ (M = Zn, Cu, Co and Ni)	20 mg/dm^3^	0.01 g/dm^3^	Xe lamp	500 W	90% (in 2 h)	[75]
TiO_2_-SnO_2_	20 mg/dm^3^	0.1 g/dm^3^	Xe lamp	150 W	90% (in 1 h)	[76]
Coumarin (C-343) Sensitized TiO_2_	40 mg/dm^3^	10–40 g/dm^3^	LED (436 nm)	5 W	-	[77]
ZnO_pH12	20 mg/dm^3^	0.1 g/dm^3^	UV-LED (365 nm)	30 W	90% (in 3 h)	[44]

**Table 6:** The photovoltaic parameters of DSSCs utilizing TiO_2_-ZnO photoanodes.

Sample	*V_OC_* (mV)	*J_SC_* (mA·cm^−2^)	*FF* (%)	*η* (%)
TiO_2_	734	13.1	67.5	6.49
TiO_2_-(2.5%)ZnO	710	15.6	71.0	7.87
TiO_2_-(5%)ZnO	764	14.2	72.9	7.90
TiO_2_-(10%)ZnO	727	14.2	71.3	7.35

**Table 7:** EIS parameters and the amounts of adsorbed dyes’ molecules of DSSCs utilizing TiO_2_-ZnO photoanodes.

Sample	*R_S_* (Ω)	*R_CE_* (Ω)	*R_CT_* (Ω)	*τ* (ms)	*N_dye_* (nmol·cm^−2^)
TiO_2_	24.8	17.4	33.3	4.1	44.3
TiO_2_-(2.5%)ZnO	21.0	6.7	20.6	7.9	43.0
TiO_2_-(5%)ZnO	16.5	6.7	22.4	6.4	40.2
TiO_2_-(10%)ZnO	18.8	6.5	27.9	10.2	38.3

## Data Availability

Not applicable.
